# Validity of handgrip strength for assessing cognition and psychotic symptoms in hospitalized patients with stable schizophrenia

**DOI:** 10.1371/journal.pone.0308133

**Published:** 2024-09-26

**Authors:** Jianlin Pu, Binyou Wang, Yilin Wang

**Affiliations:** Department of Psychiatry, Zigong Mental Health Center, The Zigong Affiliated Hospital of Southwest Medical University, Zigong, Sichuan Province, China; FEI: Centro Universitario da FEI, BRAZIL

## Abstract

**Background and objectives:**

A correlation between low handgrip strength (HGS), HGS asymmetry, and low cognitive performance has been demonstrated. However, it remains unclear whether low HGS is associated with psychotic symptoms and whether HGS asymmetry is associated with cognitive and psychotic symptoms in hospitalized patients with schizophrenia. This study aimed to investigate the validity of HGS as a measure for assessing cognition and psychotic symptoms in hospitalized patients with stable schizophrenia.

**Methods:**

A total of 235 inpatients with stable schizophrenia were recruited between August 1, 2023, and August 31, 2023. The highest HGS values from three tests on the dominant hand were used to determine low HGS (male < 28 kg, female < 18 kg), and HGS asymmetry was identified when the non-dominant HGS/dominant HGS ratio was outside 0.9–1.1. Cognition and psychotic symptoms were assessed using the Chinese Montreal Cognitive Assessment (MoCA-C) and Positive and Negative Syndrome Scale (PANSS). Generalized linear model analyses examined the relationship between HGS and scale scores.

**Results:**

Covariate-adjusted generalized linear models confirmed a strong association between low HGS alone and the MoCA-C score (OR = 0.819, 95% CI = 0.710‒0.945, p = 0.006) and PANSS score (OR = 1.113, 95% CI = 1.036‒1.239, p = 0.006). Similarly, the combination of low and asymmetric HGS was strongly associated with both MoCA-C (OR = 0.748, 95% CI = 0.653‒0.857, p<0.001) and PANSS scores (OR = 1.118, 95% CI = 1.032‒1.211, p = 0.006).

**Conclusions:**

The results suggest that hospitalized patients with schizophrenia and low HGS, with or without asymmetry, are likely to have lower MoCA-C scores and higher PANSS scores. Screening stable schizophrenia patients with low HGS, with or without asymmetry, could be a valuable and straightforward approach to identifying those with lower cognition and severe psychotic symptoms.

## Introduction

Despite advances in diagnostic and therapeutic methods, schizophrenia remains a chronic, incurable, and disabling psychiatric disorder with mechanisms that are not fully understood [1–3]. Schizophrenia is primarily characterized by psychotic symptoms and cognitive deficits [4]. In China, patients with schizophrenia are typically placed in long-term closed psychiatric hospitals or centers by their relatives or welfare institutions for management and treatment. Recent studies have shown that patients with schizophrenia in long-term care facilities are more likely to avoid social interactions, develop depression, and experience worsened cognitive impairment [5, 6]. In clinical practice, the Positive and Negative Syndrome Scale (PANSS) and the Montreal Cognitive Assessment (MoCA) are commonly used to assess psychotic symptoms and cognitive functions in schizophrenia [7–9]. However, scoring these scales requires significant time, effort, and clinical resources. Therefore, identifying a simple tool to assess mental health status and cognitive function is valuable, enabling more timely and comprehensive care.

Numerous studies have demonstrated that handgrip strength (HGS) is a reliable biomarker for assessing current conditions and future outcomes [10, 11]. HGS is a simple, non-invasive measurement easily assessed in clinical settings [12, 13]. Lack of movement or exercise, frequently observed in patients with schizophrenia in long-term care facilities, is a common cause of low HGS. Previous studies found a consistent correlation between low HGS and lower cognition in individuals with schizophrenia [14, 15]. Recent studies have shown that HGS asymmetry, defined as a non-dominant to dominant HGS ratio outside 0.9 and 1.1 [16], is associated with lower cognitive function in the general population [17, 18]. However, it remains unclear whether low HGS is associated with psychotic symptoms and whether HGS asymmetry is linked to cognitive and psychotic symptoms in hospitalized patients with schizophrenia.

To evaluate the validity of HGS as a measure for assessing cognition and psychotic symptoms in hospitalized patients with schizophrenia, this study was conducted to assist clinical practice. Patients with stable schizophrenia were recruited from a long-term closed psychiatric treatment center and categorized into four groups based on their grip strength: normal HGS, low HGS only, asymmetric HGS only, and low HGS combined with asymmetric HGS.

## Methods

### Informed consent and ethical approval process

The cross-sectional survey conducted in this research adhered strictly to the principles outlined in the Declaration of Helsinki. Two experienced psychiatrists diagnosed patients with schizophrenia based on the criteria outlined in the International Classification of Diseases, Tenth Revision (ICD-10). Patients were considered to have stable schizophrenia if their condition had remained unchanged for over a month and if they had maintained the same medication regimen for at least two months before the study commenced. A standardized protocol was used to assess participants’ ability to provide informed consent. This protocol involved a detailed assessment of their understanding of the study’s goals, methods, potential risks, and expected benefits. Participants demonstrated comprehension and the ability to make an informed decision before signing a written consent form. The consent form was designed to be clear and easy to understand, using language that was accessible to individuals with schizophrenia. If desired, participants were given ample time to review the form, ask questions, and discuss the study with a trusted individual. The entire consent process, including the capacity evaluation for stable schizophrenia patients, was thoroughly reviewed and approved by the Institutional Review Board at the Zigong Mental Health Center (IRB2023024).

### Patients’ characteristics

A prior sample size calculation was not performed in this investigation. The study aimed to enroll all patients with stable schizophrenia admitted to the Zigong Mental Health Center in China, with a potential pool of 325 individuals. The criteria for participation included: 1) being at least 18 years of age, 2) having a diagnosis of stable schizophrenia confirmed by a psychiatrist, and 3) demonstrating a willingness to participate in the study and providing informed consent. Exclusion criteria encompassed: 1) significant liver or kidney dysfunction, 2) the presence of autoimmune disorders or current anti-cancer therapy, 3) lack of a diagnosis of stable schizophrenia, 4) incomplete HGS testing or scale assessments, and 5) failure to sign an informed consent form. The research was executed between August 1, 2023, and August 31, 2023.

### Low HGS and HGS asymmetry assessment

Participants’ HGS was measured while standing with the elbow vertical to the ground, ensuring a small gap between the arm and the body. Measurements were taken using a dynamometer (EH101; Camry, Guangdong, China) maintained under standardized conditions by trained technicians. The dynamometer ranges from 0 to 90.0 kg. Testing began with the non-dominant hand, followed by the dominant hand. After each grip strength test cycle, participants rested for 2 minutes before starting the next cycle. Testing was conducted three times per hand. According to the consensus of the Asian Working Group for Sarcopenia (AWGS) 2019 [19], HGS was analyzed using the maximum value of three repetitions from the dominant hand. Low HGS was defined as < 28 kg for males or < 18 kg for females. HGS asymmetry was defined as a non-dominant to dominant HGS ratio greater than 1.1 or less than 0.9 [17, 20].

### Cognition and psychotic symptoms assessment

The MATRICS Consensus Cognitive Battery (MCCB) was crafted as the primary assessment instrument for clinical trials to enhance cognitive functions in individuals with schizophrenia [21]. Its development was driven by the necessity for a robust tool that could effectively evaluate the efficacy of various interventions on cognitive performance within this patient population. The MCCB has since undergone extensive testing to establish its reliability and validity in gauging cognitive abilities in those with schizophrenia, and it has gained widespread adoption in the field [22–25]. However, constrained by the expertise of the staff involved in the present study and the logistical limitations, including hardware, software, and time constraints for comprehensive cognitive assessment, we elected to utilize the Chinese version of the Montreal Cognitive Assessment (MoCA-C) for our cognitive screening in schizophrenia patients [26]. Current research validates the concurrent total score correlation between the MoCA and the MCCB, underscoring the MoCA’s utility as a screening tool for cognitive impairment in schizophrenia patients, particularly due to its ease of use and brief administration time [8, 9, 27]. The MoCA-C scale requires completion within 15 minutes, with a maximum score of 30 points. A lower score indicates poorer cognitive function.

The psychotic symptoms of the patients were evaluated by expert psychiatrists using the PANSS [7, 28]. The PANSS consists of 30 items, with each item rated on a scale of 1 to 7 based on the severity of symptoms. Higher ratings indicate more pronounced or severe symptoms.

### Covariates

Covariate information was gathered through self-reports and data extracted from electronic medical records. This included all relevant details available within the electronic medical records for analytical purposes. Information included age, sex, height, weight, disease duration, hospitalization time, number of siblings, number of children, family history of mental disorder, first episode, marital status (married or unmarried/divorced/widowed), education (illiterate, high school and below, university and above), vision problems, hearing problems, smoking history, drinking history, falls history, COVID-19 history, number of chronic diseases, antipsychotics (typical, atypical, combined), and anxiety and depression status. The severity of anxiety symptoms and functional effects in the past two weeks were assessed using the Generalized Anxiety Disorder 7 (GAD-7), while the severity of depression was evaluated using the Patient Health Questionnaire-9 (PHQ-9). Scores below 5 on these scales indicate no anxiety or depression.

The covariate data for each patient were meticulously collected individually before proceeding to the next patient. Once all necessary covariates, excluding height, weight, PHQ-9 scores, and GAD-7 scores, had been obtained from the electronic medical records, the research team initiated the measurement of each patient’s height and weight. Immediately after that, the PHQ-9 and GAD-7 assessments were administered to the patients. This entire process was overseen by trained research staff, who were dedicated to ensuring the accurate measurement of height and weight, as well as the proper administration and scoring of the assessments, to guarantee a thorough and precise data collection for every patient. As a result, covariate data for all included patients were successfully collected without missing values, obviating the need for specific actions to address missing data.

### Statistical analyses

The data were analyzed using SPSS version 25.0, with statistical significance set at a two-tailed p-value < 0.05. Categorical variables are presented as frequencies with corresponding percentages. Quantitative variables, including age, BMI, duration of disease, hospitalization time, number of siblings and children, GAD-7 scores, and PHQ-9 scores, were categorized as follows: age (<60 or ≥60 years), disease duration (<5 or 5–10 or >10 years), hospitalization time (<6 or ≥6 months), number of siblings (≤1 or ≥2), number of children (0 or ≥1), GAD-7 (<5 or ≥5), PHQ-9 (<5 or ≥5), and BMI (<18.5, 18.5–23.9, or ≥24). Due to the small number of individuals with a BMI <18.5 (n = 8), this group was combined with the 18.5–23.9 group, resulting in BMI categories of <24 and ≥24. Subgroups were also described with median values, accompanied by the 25th (P25) and 75th (P75) percentiles, for a clearer understanding of variable distribution.

Patient characteristics across different groups were compared using non-parametric rank-sum tests for two or more independent samples. Similarly, patient MoCA-C and PANSS scores between abnormal HGS groups were compared using non-parametric rank-sum tests. A modified Poisson regression analysis was employed to evaluate the potential relationship between abnormal HGS and scale scores. We generated two models. Model 1 was unadjusted. For further adjustment (Model 2), covariates demonstrating a significant association with scale scores (p < 0.05) were included. These covariates included age, sex, marital status, education, hearing problems, smoking history, falls history, COVID-19 history, and the number of chronic diseases for the MoCA-C score. Additionally, sex, hearing problems, and GAD-7 were included as covariates for PANSS scores.

## Results

Among the 325 patients admitted, 12 were under 18, 20 had severe liver or kidney diseases or were undergoing anti-cancer therapy, 20 had not completed at least two grip strength tests, 8 were not in stable condition, and 30 chose not to participate. Consequently, data from 235 patients meeting the inclusion and exclusion criteria were collected and analyzed.

Table 1 summarizes the characteristics of inpatients with stable schizophrenia. A total of 235 patients with stable schizophrenia were enrolled, consisting of 60% males (141/235) and 40% females (94/235). The MoCA-C and PANSS scores among participants were 18 (12, 23) and 62 (52, 72), respectively. Among inpatients, 90.2% were under 60 years old, 54.9% had a BMI over 24, 74.9% had a disease duration over 10 years, 83.4% were hospitalized over 6 months, 86% had more than 2 siblings, 51.5% had no children, 22.6% had a family history of mental disorder, 3% were first-episode, 80.4% were unmarried/divorced/widowed, 90.6% had an education level of illiterate or high school and below, 13.2% had vision problems, 7.2% had hearing problems, 41.7% had a smoking history, 25.5% had a drinking history, 6% had a history of falls, 40.0% had a history of COVID-19, 32.3% had a chronic disease, 28.1% had depression, 15.3% had anxiety, and 97.9% used atypical or combined antipsychotics.

**Table 1:** Participant characteristics of inpatients with stable schizophrenia.

Variable	N(%)	MoCA-C scores,median (P25,P75)	P-value	PANSS scores,median (P25,P75)	P-value
**Age,years, median (P25,P75)**			**0.005**		0.304
All, 51(41,56)	235(100)	18(12,23)		62(52,72)	
<60,49(39,54)	212(90.2)	18.5(12.25, 24)		62(51.25,71)	
≥60,65(61,68)	23(9.8)	13(7,18)		64(57,75)	
**BMI,kg/m** ^**2**^ **, median (P25,P75)**			0.06		0.658
<24,21.75(20.08,23.21)	106(45.1)	19.5(14,24)		62.5(51.75,72)	
≥24,27.28(25.47,29.34)	129(54.9)	17(11,23)		62(52,71.5)	
**Disease duration, years, median (P25,P75)**			0.203		0.099
<5, 3(2.5,4)	13(5.5)	20(13.5,22.5)		60(52,67)	
5–10, 9(6.75,10)	46(19.6)	20(14.75,23.25)		57(50,65.25)	
>10, 22.5(17,30.75)	176(74.9)	17(11,23.75)		64(52,74)	
**Hospitalized time, months, median (P25,P75)**			0.168		0.487
<6, 3(2,4)	39(16.6)	20(13,24)		64(55,70)	
≥6,32.5(16,60.75)	196(83.4)	18(12,23)		62(51,72)	
**Sex**			**0.001**		**0.002**
Male	141(60)	20(14,24)		59(50,69)	
Female	94(40)	16(9,21)		65(56,76)	
**Number of siblings, median (P25,P75)**			0.141		0.956
≤1, 1(0,1)	33(14.0)	20(15,24)		63(53,70)	
≥2, 3(2,4.25)	202(86.0)	18(11,23)		62(51,72)	
**Number of children, median (P25,P75)**			0.112		0.849
0, 0(0,0)	121(51.5)	20(12,24)		62(52,70)	
≥1, 1(1,2)	114(48.5)	17(11,22)		63(50.75,74)	
**PHQ-9 Scores, median (P25,P75)**			0.917		0.122
<5, 1(0,3)	169(71.9)	18(11,24)		62(51,70)	
≥5, 7(6,9)	66(28.1)	18(13.75,23)		65(55,75.25)	
**GAD-7 Scores, median (P25,P75)**			0.573		**0.048**
<5, 0(0,2)	199(84.7)	18(12,24)		62(51,70)	
≥5,6.5(5,7.75)	36(15.3)	17(11,22)		65(56.25,79.5)	
**Family history of mental disorder**			0.954		0.826
No	182(77.4)	18(12,23.25)		62(52,71)	
Yes	53(22.6)	19(11,23)		63(51.5,73.5)	
**First episode**			0.637		0.78
No	228(97)	18(12,23)		62(52,72)	
Yes	7(3)	21(14,23)		67(48,70)	
**Marital status**			**0.007**		0.865
Married	46(19.6)	15(9.5,20)		64(52,72)	
Unmarried/divorced/ widowed	189(80.4)	19(12,24)		62(51.5,71.5)	
**Education**			**<0.001**		0.068
Illiterate	16(6.8)	7(4.25,13)		63.5(57.25,69)	
High school and below	197(83.8)	18(12,23)		63(52,74)	
University and above	22(9.4)	24(21,27)		53.5(47.25,66)	
**Vision problems**			0.107		0.628
No	204(86.8)	17(11,23)		62.5(52,71)	
Yes	31(13.2)	21(17,24)		62(50,80)	
**Hearing problems**			**0.007**		**0.027**
No	218(92.8)	18(12,24)		60.5(51.0,71.0)	
Yes	17(7.2)	13(7,18)		69(63.5,76)	
**Smoking history**			**0.049**		0.234
No	137(58.3)	17(11,22)		63(52,74)	
Yes	98(41.7)	19(14,24)		62(49.75,70)	
**Drinking history**			0.566		0.804
No	175(74.5)	18(12,24)		62(52,71)	
Yes	60(25.5)	17(12.25,22.75)		63.5(50.25,73.5)	
**Falls history**			**0.001**		0.108
No	221(94.0)	18(12.5,23.5)		62(51.5,71)	
Yes	14(6.0)	10.5(6.25,13.75)		68.5(58,80.25)	
**COVID-19 history**			**0.01**		0.967
No	141(60.0)	19(13,24)		63(52,70.5)	
Yes	94(40.0)	17(9,21)		61.5(51,74.25)	
**Number of chronic diseases**			**0.044**		0.122
0	159(67.7)	19(13,24)		59(51,70)	
1	40(17.0)	16(9.25,20.75)		64.5(50.75,74.25)	
≥2	36(15.3)	16(9.5,21)		66(54,77.5)	
**Anti-psychotics**			0.488		0.725
Typical	5(2.1)	24(13,25.5)		53(46,75)	
Atypical	214(91.1)	18(12,23)		63(52,72)	
Combined	16(6.8)	21(9.5,24.75)		59.5(50.75,74.5)	

Note: GAD-7 = Generalized Anxiety Disorder; PHQ-9 = Patient Health Questionnaire-9; MoCA-C = Montreal Cognitive Assessment-Chinese version; PANSS = Positive and Negative Syndrome Scale.

Furthermore, the age was divided into two groups: <60 years old and ≥60 years old. The medians (P25, P75) for these groups were 49 (39, 54) and 65 (61, 68) respectively. The BMI (kg/m^2^) was also divided into two groups: <24 and ≥24. The medians (P25, P75) for these groups were 21.75 (20.08, 23.21) and 27.28 (25.47, 29.34) respectively. The disease durations (years) were divided into three groups: 5, 5–10, and over 10. The medians (P25, P75) for these groups were 3 (2.5, 4), 9 (6.75, 10), and 22.5 (17, 30.75) respectively. The hospitalized time was divided into two groups: <6 months and ≥6 months. The medians (P25, P75) for these groups were 3 (2, 4) and 32.5 (16, 60.75) respectively. The GAD-7 scores were divided into two groups: <5 and ≥5. The medians (P25, P75) for these groups were 0 (0, 2) and6.5 (5, 7.75) respectively. Lastly, the PHQ-9 scores were also divided into two groups: <5 and ≥5. The medians (P25, P75) for these groups were 1 (0, 3) and 7 (6, 9) respectively. Significant differences were observed in age (p = 0.005), sex (p = 0.001), marital status (p = 0.007), education level (p<0.001), hearing problem (p = 0.007), smoking history (p = 0.049), history of falls (p = 0.001), history of COVID-19 (p = 0.01), and number of chronic diseases (p = 0.044) when comparing the MoCA-C scores. However, no differences were found for other covariates that were analyzed. Similarly, significant differences were found in sex (p = 0.002), hearing problem (p = 0.027), and GAD-7 scores <5 or ≥5 (p = 0.048) when comparing the PANSS scores, with no differences observed in other covariates.

Table 2 presents univariate analysis results for HGS groups and scale scores. Patients with HGS asymmetry alone, low HGS alone, and low HGS with asymmetry were 27.2%, 19.6%, and 28.1%, respectively. MoCA-C and PANSS scores were 21 (14.25, 24) and 56 (50.25, 67.75) for patients with HGS asymmetry alone, 17.5 (8.75, 22.25) and 67.5 (55, 76.25) for patients with low HGS alone, and 14 (8, 19.25) and 66 (58.5, 76) for patients with weak and asymmetric HGS, respectively. Significant differences in MoCA-C scores (p < 0.001) and PANSS scores (p = 0.001) were observed among different HGS groups.

**Table 2:** Univariate analysis of handgrip strength and scale scores.

Variable	n(%)	MoCA-C scores,	P-value	PANSS scores,	P-value
		median (P25,P75)		median (P25,P75)	
Normal HGS	59(25.1)	20(16,25)	<**0.001**	59(46,69)	**0.001**
Asymmetric HGS only	64(27.2)	21(14.25,24)		56(50.25,67.75)	
Low HGS only	46(19.6)	17.5(8.75,22.25)		67.5(55,76.25)	
Low and asymmetric HGS	66(28.1)	14(8,19.25)		66(58.5,76)	

Note: MoCA-C = Montreal Cognitive Assessment-Chinese version; PANSS = Positive and Negative Syndrome Scale.

Table 3 presents the correlation results between HGS and scale scores in patients with stable schizophrenia. After adjusting for potential confounders such as age, sex, marital status, education, hearing problem, smoking history, falls history, COVID-19 history, and number of chronic diseases, it was found that HGS weakness alone (OR = 0.819, 95%CI = 0.710–0.945, p = 0.006) and weak HGS with asymmetry (OR = 0.748, 95%CI = 0.653–0.857, p<0.001) were strongly associated with decreased scores on the MoCA-C. Similarly, after adjusting for sex, hearing problem, and GAD-7 scores (<5/≥5), it was observed that low HGS alone (OR = 1.113, 95%CI = 1.036–1.239, p = 0.006) and low HGS with asymmetry (OR = 1.118, 95%CI = 1.032–1.211, p = 0.006) showed a strong association with increased scores on the PANSS.

**Table 3:** Analysis of the correlation between handgrip strength and scale scores.

Variable	MoCA-C			PANSS		
	χ2	P-value	OR(95%CI)	χ2	P-value	OR(95%CI)
Normal HGS	-	-	1	-	-	1
Asymmetric HGS only	0.192	0.662	0.976(0.877,1.087)	0	0.987	0.999(0.920,1.086)
Low HGS only	7.509	0.006	0.819(0.710,0.945)	7.523	0.006	1.113(1.036,1.239)
Low and asymmetric HGS	17.541	<0.001	0.748(0.653,0.857)	7.499	0.006	1.118(1.032,1.211)

Note: HGS = handgrip strength; MoCA-C = Montreal Cognitive Assessment-Chinese version; PANSS = Positive and Negative Syndrome Scale. MoCA-C: adjusting for age, sex, marital status, education, hearing problem, smoking history, falls history, COVID-19 history, and number of chronic diseases. PANSS: adjusting for sex, hearing problem and GAD-7 scores (<5 / ≥5).

## Discussion

This study is the first to investigate the validity of HGS as a measure for assessing cognition and psychotic symptoms in hospitalized patients with schizophrenia in long-term care facilities. Results confirmed that HGS can be an additional tool for assessing cognitive and mental health in hospitalized patients with stable schizophrenia. Specifically, patients with only asymmetric HGS showed no association with MoCA-C and PANSS scores, while patients with low HGS, with or without asymmetry, were closely associated with MoCA-C and PANSS scores. Based on these findings, it is recommended to screen for abnormal HGS, especially in patients with low HGS, with or without asymmetry, as these individuals typically have lower cognition and more severe symptoms.

Previous studies have shown that HGS is influenced by age [29, 30], increasing until the age of 30 [31] and then declining from 40 in women and 50 in men [32]. As individuals age, factors such as decreased physical performance, motoric cognitive risk syndrome, brain hemisphere dysfunction, and deficits due to acute and chronic injuries gradually appear. These factors are possible mechanisms associated with HGS asymmetry [33–35]. Therefore, it is common for older individuals to have low or asymmetric HGS [16, 33, 36, 37].

A previous study involving 10,563 community-dwelling Chinese individuals aged 45 and above reported that 12.47% had low HGS only, 27.15% had HGS asymmetry only, and 14.28% had both low HGS and asymmetry [38]. In this study, patients with schizophrenia aged 51 (41, 56) years were enrolled. Similar proportions were observed, with 27.2% exhibiting HGS asymmetry but significantly higher proportions with 19.6% and 28.1% exhibiting low HGS only and low HGS with asymmetry. These differentiated results may be attributed to the following factors: 1) Schizophrenia accelerates the aging process and causes body functions to decline faster than in normal individuals [39]; 2) Comorbidities associated with schizophrenia and the use of different medications worsen physical fitness compared to healthy individuals [40–42]; 3) Living in a closed long-term care facility leads to reduced activity and exercise compared to peers.

Consistent with previous studies [14, 15], this study also observed a correlation between low HGS and lower cognition in individuals with schizophrenia. Additionally, a strong correlation between low HGS and severe symptoms was found in this study. However, no relationship between HGS asymmetry alone and cognition or psychotic symptoms was found among these patients. Patients with both low and asymmetric HGS showed a strong correlation with lower cognition and more severe symptoms. Patients with low HGS, with or without asymmetry, had significantly lower cognitive function scores and higher psychotic symptoms scores. Taken together, these results suggest that low HGS, with or without asymmetry, can be used as an additional tool to identify hospitalized patients with stable schizophrenia who have lower cognitive function and more severe symptoms.

The current study has several limitations that should be acknowledged. Firstly, as an observational study, it may be affected by biases such as the inability to establish causal relationships and potential oversight of certain confounding variables. Secondly, the sample size was small and limited to one long-term care facility, raising concerns about the generalizability of the findings. Thirdly, the scales were scored by two psychiatrists, and despite their specialized training, there may still be inconsistencies in the scoring process. Additionally, using the MoCA instead of the MCCB for cognitive assessment, despite their strong correlation, may introduce subtle discrepancies in evaluating certain items. To overcome these limitations, future research should consider employing more sophisticated designs, such as cohort studies, to address causality, expanding sample sizes and recruitment to enhance generalizability, standardizing scoring procedures to minimize inconsistencies, and comparing the MoCA and MCCB to identify discrepancies in cognitive assessment.

## Conclusions

In conclusion, the present study suggests that hospitalized schizophrenia with low HGS with or without asymmetry, are highly likely to have lower MoCA scores and higher PANSS scores. Screening for patients with stable schizophrenia who exhibit low HGS with or without asymmetry could be a valuable and straightforward approach to evaluate those patient populations with lower cognition and severe psychotic symptoms, which in return may contribute to improved timely treatment and careless for them.
